# Managing Pulmonary Complications in Adults With Cerebral Palsy: A Vulnerable Transition

**DOI:** 10.1016/j.chpulm.2023.100025

**Published:** 2023-10-10

**Authors:** Brinda Desai, Daniel J. Lesser, Bernie Y. Sunwoo

**Affiliations:** a Division of Pulmonary, Critical Care, Sleep Medicine & Physiology, University of California San Diego, La Jolla; b Division of Respiratory Medicine, Rady Children’s Hospital San Diego, UC San Diego Department of Pediatrics, San Diego, CA

**Keywords:** cerebral palsy, CP, pulmonary complications in cerebral palsy, pulmonary complications in CP, transition in cerebral palsy, transition in CP

## Abstract

Cerebral palsy is the most common motor disability in childhood with increasing survival rates into adulthood. Pulmonary complications are a leading cause of morbidity and mortality in this population. However, adult pulmonologists are rarely trained on how to receive these patients into their practice, including how best to identify and manage pulmonary complications. Quality studies on pulmonary complications in cerebral palsy, both in the adult and pediatric population, are lacking, but we review the available literature to provide an approach for the adult pulmonologist in identifying risk factors for pulmonary complications including aspiration, impaired airway clearance, airways disease, neuromuscular scoliosis and restrictive chest wall disease, sleep-disordered breathing, and hypoventilation. We provide a framework to help manage these pulmonary complications and plan and organize successful multidisciplinary transition of pulmonary care from adolescence to adulthood, largely extrapolating from small pediatric studies, studies on neurodevelopmental disorders in general, international guidelines, and clinical experiences, until more quality studies on this topic are available.

Cerebral palsy (CP) describes a group of permanent disorders of the development of movement and posture that are attributed to nonprogressive disturbances occurring in the developing fetal or infant brain.^1^ It is the most common motor disability of childhood, and survival rates have improved with almost all children with CP surviving into adulthood.^2^ The clinical manifestations of CP are variable, and often accompanied by disturbances of sensation, cognition, behavior, seizures, and secondary musculoskeletal complications, but pulmonary complications are a leading cause of morbidity and mortality. The risk of death from pulmonary causes is 14 times higher in adults with CP than adults with no disability.^3^

Transition of medical care from pediatrics to adults is a vulnerable period, with many young adults with CP and their caregivers reporting difficulties navigating the adult health care system, difficulties obtaining subspecialized care, and disruptions to continuity of care.^4^ Given the rising prevalence of adult patients with CP and the pulmonary complications these patients often incur (Fig 1), we find it important to understand how pulmonary complications present in this population and available treatment modalities. However, there are few clinical studies currently to guide physicians on the pulmonary care of adults with CP. Guidelines are emerging.^5^, ^6^, ^7^ A care pathway with recommendations for assessment and treatment of pulmonary complications in children with CP has been developed by the American Academy for Cerebral Palsy and Developmental Medicine (Fig 2),^8^ and many of the suggestions are applicable to adults.^5^, ^6^, ^7^

**Figure 1:**
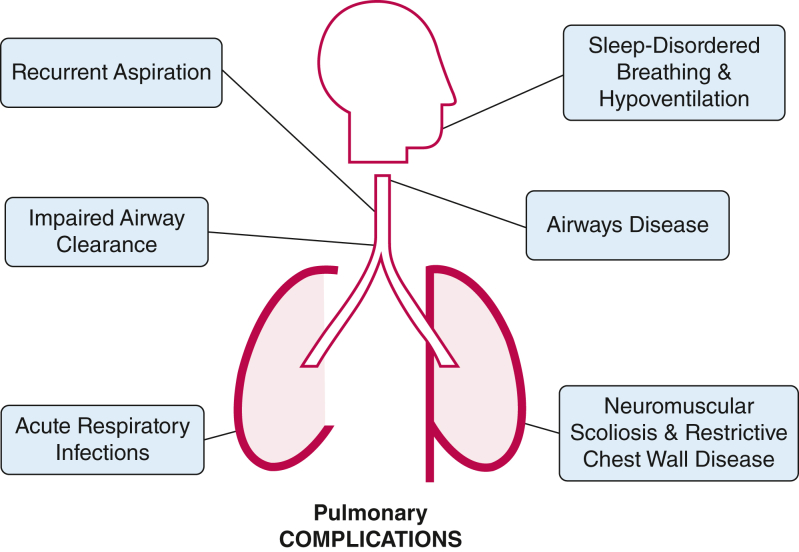
Pulmonary complications associated with cerebral palsy.

**Figure 2:**
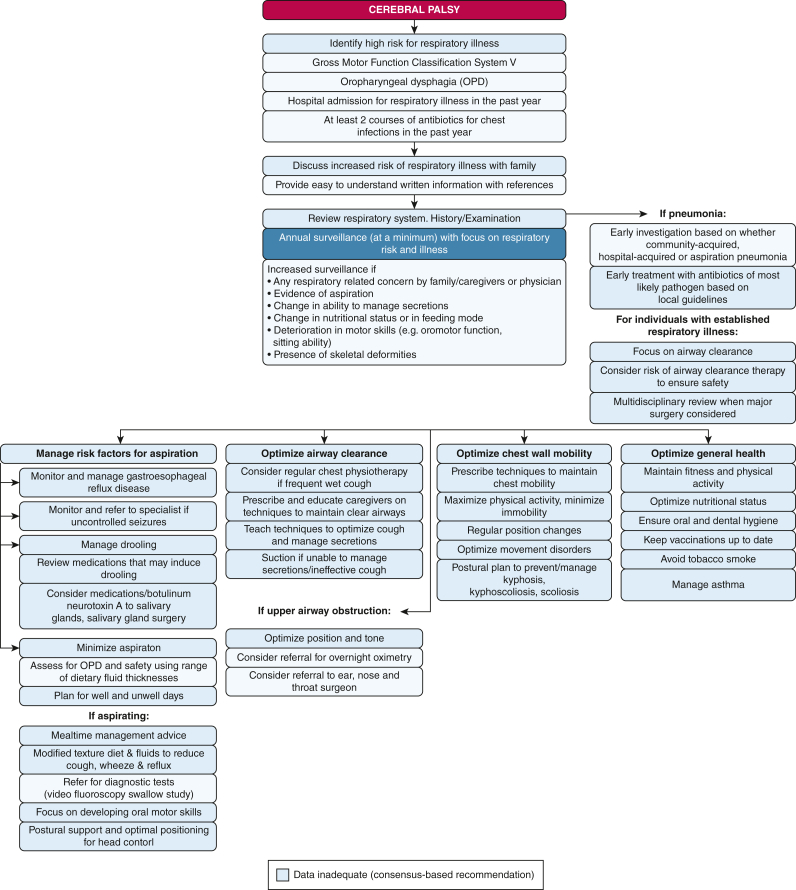
Flow diagram for evidence-informed clinical practice guidelines for individuals with cerebral palsy and high risk of respiratory illness. (Adapted with permission from the American Academy of Cerebral Palsy and Developmental Medicine Respiratory Health in Cerebral Palsy Care Pathway^5^^,^^8^). OPD = oropharyngeal dysphagia.

## Case

A 21-year-old woman with spastic CP and hydrocephalus status post-ventriculoperitoneal shunt was followed by a pediatric pulmonologist for chronic respiratory failure attributed to recurrent aspiration status post-gastrostomy, scoliosis status post-spine stabilization, and severe OSA on bilevel positive airway pressure. She presents to the adult pulmonary clinic in a wheelchair, accompanied by her father, stating she was told she would now need to be followed by an adult pulmonologist after losing health insurance coverage. She had some cognitive impairment but her father was able to provide additional history.

This case highlights pulmonary considerations in the transition of a patient with CP from pediatric to adult medicine including the following: (1) multidisciplinary care coordination, (2) aspiration, (3) impaired airway clearance, (4) airways disease, (5) neuromuscular scoliosis and restrictive chest wall disease, and (6) sleep-disordered breathing (SDB) and hypoventilation.

## Recognizing Pulmonary Complications in CP

**Figure 3:**
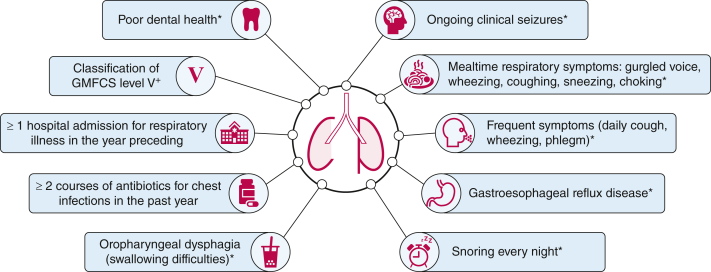
Identified risk factors in cerebral palsy associated with pulmonary complications and hospital admissions. Young people with cerebral palsy who have any of these risk factors are more likely to have at least one hospital admission for chest infections in the next 5 y.^9^^,^^10^^+^Gross Motor Classification System level V: difficulty controlling head and body position in most positions. ∗Modifiable risk factors. GMFCS = Gross Motor Function Classification System.

Blackmore et al^9^^,^^10^ identified nine risk factors for respiratory hospitalizations in a prospective cohort of children and young adults with CP followed for 5 years (Fig 3). Furthermore, Gibson et al^11^ stress the importance of dental hygiene in patients with CP because bacteria in the mouth can lead to respiratory complications. At the initial visit, we focus on identifying these nonmodifiable and modifiable risk factors for pulmonary complications through a detailed history and physical examination, including review of pediatric medical and dental records. Pulmonary complications are related to CP severity. Several classification systems exist to grade impairments associated with CP, and the Gross Motor Function Classification System (GMFCS) is most commonly used (Table 1).^12^ GMFCS classifies children into five levels of motor function up to 18 years of age with level V requiring a wheelchair in all settings. GMFCS level V is the strongest predictor of pulmonary complications including hospitalizations for pulmonary infections.^9^, ^10^, ^11^^,^^13^ We identified multiple risk factors for pulmonary complications in the presenting patient, including GMFCS level V classification, frequent respiratory symptoms, oropharyngeal dysphagia, and gastroesophageal reflux (GER) disease, for which she had already undergone fundoplication and gastrostomy with strictly no oral intake.

**Table 1:** Gross Motor Function Classification System - Expanded & Revised

GMFCS - E&R Level	GMFCS - E&R Description
I	• Can walk independently in multiple environments including home, school, and outdoors. • Can climb stairs without the use of a railing or assistance. • Can perform gross motor skills (eg, running, jumping) but limited in terms of speed, balance, and coordination.
II	• Can walk in most settings independently but requires the use of railings when climbing stairs. • Difficulty walking long distances, on inclines, or uneven surfaces. • Minimal ability to perform gross motor skills (eg, running, jumping).
III	• Needs the assistance of a mobility device in most settings. • May be able to climb stairs with physical assistance or by holding onto rails.
IV	• Requires physical assistance or dependent on powered mobility devices in most environments.
V	• Requires a manual wheelchair with a caregiver for transportation in all settings. • May be limited in ability to maintain antigravity positions. • May be limited in control of head, trunk, arms, and legs.

Five-level classification system that describes the gross motor function of children and youth (between 2 and 18 y of age) with cerebral palsy based on sitting, walking, and wheeled mobility. Distinctions between levels are based on functional abilities, need for assistive technology, and to a lesser extent, quality of movement.^12^ GMFCS - E&R = Gross Motor Function Classification System - Expanded & Revised.

## Multidisciplinary Care Coordination

Transition has been defined by Blum et al^14^ as the “purposeful, planned movement of adolescents and young adults with chronic physical and medical conditions from child-centered to adult-oriented health care systems.” Several documents are available to guide practitioners and health care systems seeking to transition health care from adolescence to adulthood. Got Transition^15^ is a US nationally funded resource center and website aiming to improve transition by working with physicians, health programs, payers, young adults, and caregivers. They recommend establishing a process for integrating core elements necessary for successful transition, including development and implementation of transition policies, monitoring progress in transition goals, assessment of readiness, development of a health care plan with a medical summary for the adult center, transfer of care, and confirmation of transfer completion.^15^ The importance of both pediatric and adult centers acting in partnership at the individual, departmental, and institutional levels cannot be understated.

Although guidelines to standardize transition have existed for several years, patients, caregivers, and practitioners still face barriers. There is often loss of access to comprehensive health insurance coverage and government funded programs with age.^16^ The presenting patient had been receiving California Children Services, a public state program that provides diagnostic and treatment services, medical case management, and physical and occupational therapy services to children with certain chronic medical conditions including CP, but at 21 years of age she was no longer eligible. Planning is necessary to minimize the disruption and confusion created by abrupt ends in health insurance coverage. Discussions should start in early adolescence and be ongoing. Having an adult partner who specializes in or is open to becoming acquainted with CP and its pulmonary management can be crucial. For the patient, transition occurred between health care systems in the same city using the same electronic medical records system that allowed immediate review of medical records. Additionally, the pediatric and adult pulmonologists had contact details for each other, and in our experience, direct communication between the pediatric and adult physicians and team members is invaluable. Social workers and financial counselors can help navigate changes in insurance coverage. Nursing or respiratory therapists can help manage issues surrounding changes in durable medical equipment vendors and medical equipment that often accompany insurance changes. Unfortunately, access to these resources is often limited. In hospitals caring for adults, equipment commonly used in children’s hospitals (eg, pediatric-sized tracheostomy tubes) may not be readily available. Staff may possess varying levels of experience in managing adults with complex medical needs (eg, CP). Frank conversations can acknowledge differences in resources and the imperfections in the transition process. A pledge to support the patient with equipment ordering, medication prescriptions, and health advice until transition is complete can inspire confidence for patients and their families.

## Aspiration

Patients with CP have multiple risk factors for aspiration including oropharyngeal dysphagia, drooling, GER, and epilepsy causing vomiting. Aspiration is reported in 26% to 70% of children and adults with CP, and can lead to recurrent bacterial infections or chronic inflammatory responses in the lungs.^17^^,^^18^ We ask about any history of coughing, choking, or gagging when eating and drinking; frequent chest infections; and lengthy meal duration, but aspiration can be silent. Therefore, a high degree of clinical suspicion is warranted.

We suggest early involvement of speech pathology when aspiration is suspected. Fiberoptic endoscopic evaluation of swallowing and videofluoroscopy can be considered if there is ongoing uncertainty about the safety of swallowing after clinical assessment, if there are recurrent chest infections without obvious clinical signs of aspiration, if there is deterioration in feeding and drinking ability, and to aid in prognostication.^19^ Testing may be limited by intellectual and motor disabilities. In the presence of oromotor dysfunction, optimizing posture and position and speech pathology input regarding dietary modifications can minimize aspiration risk. If swallowing difficulties persist, placement of gastrostomy tubes may be considered, as was done in the presenting patient, especially if struggling to meet nutritional requirements. However, gastrostomy tubes, including postpyloric tubes, offer no protection from colonized oral secretions, and scintigraphic studies have demonstrated aspiration of gastric contents in patients fed by gastrostomy tubes.^20^ Studies on gastrostomy tubes in CP have yielded mixed results in terms of decreasing respiratory infections.^21^ The relationship between GER and pulmonary complications is complex and poorly understood.^17^ Although GER is common in CP, assessing for classic symptoms of GER can be challenging in CP. Invasive diagnostic testing including upper endoscopy and ambulatory esophageal pH with impedance monitoring is available but not without its own challenges in this population. Treatment is usually multimodal incorporating dietary and lifestyle modifications, medical therapies, and surgery, but the impact of these therapies on pulmonary outcomes in CP is unclear.^21^^,^^22^ The presenting patient had undergone fundoplication.

When assessing aspiration risk, drooling should be evaluated. Drooling is common in CP and can be severe. It too is caused by poor oromotor function, and not hypersalivation.^23^ Review of postural support including head control and equipment for suctioning are essential. Anticholinergic medications can reduce the severity and frequency of drooling, but data regarding the effect on respiratory status are missing.^18^^,^^19^^,^^24^ Use of anticholinergics is often limited by side effects, including dry mouth, thickened secretions, and urinary retention, with glycopyrrolate potentially having fewer side effects than other anticholinergic agents.^25^ The presenting patient was receiving glycopyrrolate with good effect and reported no side effects. The medication was renewed for her. If medications prove unsuccessful, salivary gland botulinum toxin injections or surgery may be options.^11^^,^^17^^,^^18^^,^^26^^,^^27^

## Airway Clearance

Mucociliary clearance and cough normally work together to clear aspiration of orogastric contents. These normal host defenses are often impaired in CP, especially in patients with severe motor impairment, increasing risk of respiratory infections. There is biological plausibility for use of airway clearance techniques (ACTs) in preventing and managing respiratory infections in CP. However, very few studies are available on ACTs and respiratory outcomes in patients with CP.

Multiple ACTs are available that vary in their physiological effects and assistance with sputum expectoration (Table 2).^28^^,^^29^ The best ACT method(s) and frequency in CP are unknown. Small studies in CP have suggested a possible benefit in reducing hospitalizations with higher adherence to high-frequency chest wall oscillation devices and mechanical insufflation-exsufflation.^17^^,^^29^^,^^30^ In our experience, most patients are on ACTs at the time of transition, but as patients mature, ACTs should be individualized to the patient’s symptoms, mobility, cognition, caregiver availability, and insurance/costs.^5^ A multidisciplinary approach with a respiratory physiotherapist or respiratory therapist with specialized knowledge and experience in CP is best.^5^ The presenting patient who had limited mobility, reduced cognition, and a weak cough reported using high-frequency chest wall oscillation or vest therapy midafternoon and a mechanical insufflator-exsufflator in the evenings. With this ACT regimen, she had not had recent hospitalizations or antibiotic use, and she was connected with new durable medical equipment covered by her new health insurance for ongoing equipment. Additionally, pulmonary rehabilitation and exercise training should be considered with evidence from uncontrolled studies that exercise may improve pulmonary function in children with CP; however, improvements in respiratory health have not yet been shown.^17^^,^^31^

**Table 2:** Airway Clearance Modalities

Airway Clearance Technique	Mechanism	Advantages	Disadvantages
Manual chest physiotherapy	Manual percussion is applied over various parts of the thorax in a rhythmic manner to promote mobilization of secretions.	Low cost. Useful for patients unable to cooperate with therapy based on cognitive or muscular impairment.	Difficult for patients with deformity of chest wall and/or back, or ineffective cough. Requires physical assistance.
Gravity-assisted/postural drainage	Having patients assume various positions to promote maximal drainage of secretions in the direction of gravity.	Low cost.	Difficult for patients with significant cognitive impairment, deformity of chest wall and/or back, or ineffective cough. Requires physical assistance.
Intrapulmonary percussive ventilation device	Delivers high-flow, low-volume positive pressure to the airway at varying frequencies, often in conjunction with a bronchodilator.	Useful in patients with asthma or reactive airway disease. Can be self-managed.	Can be costly. Difficult for patients with significant cognitive impairment. Device may not be easy to transport.
Positive expiratory pressure devices	Uses a one-way valve that allows unrestricted inspiration and a resistance to expiration.	Easy to transport. Can be self-managed.	Difficult for patients with significant cognitive impairment or ineffective cough.
High-frequency chest wall oscillations (“vest” therapy)	High-frequency air pulses are delivered to the device which causes oscillatory chest wall compressions every few minutes for about 20-30 min.	Easy to use. Can be self-managed.	Can be costly. Difficult for patients with ineffective cough. Cannot be used if recent chest trauma. Device may not be easy to transport.
Mechanical insufflation/exsufflation	Delivers positive-pressure insufflation followed by an expulsive exsufflation.	Good for patients with an ineffective cough.	Can be costly. Cannot be used if recent chest or maxillofacial trauma. May not be used if recent abdominal surgery. Requires training.

Various adjunct airway clearance modalities are shown in addition to exercise and breathing techniques that can be considered in clinical practice based on extrapolation from literature evaluating effectiveness in bronchiectasis.^28^ Different airway clearance techniques can be combined with each other (eg, oscillatory positive expiratory pressure can be combined with high-frequency chest wall oscillations).^29^

Mucoactive agents (eg, hypertonic saline) potentially increase the ability to expectorate sputum and/or decrease mucus hypersecretion, and are often bundled together with ACTs.^32^ The presenting patient was not on a mucoactive agent at the time of transition. There is no high-quality evidence around the use of mucoactive agents in CP as an adjunct to ACTs, and most recommendations are extrapolated from the bronchiectasis literature where studies are mixed.^13^^,^^22^^,^^29^ Recombinant human deoxyribonuclease has been associated with more frequent exacerbations and FEV_1_ decline in noncystic fibrosis bronchiectasis, and its use in CP is cautioned.^13^^,^^33^ It is our observation that mucoactive agents are commonly used as adjuncts to ACTs, but until further studies prove efficacy, it is important to monitor for clinical benefits of these therapies, especially because they can add significant burden of care and cost to patients and their families.

## Airways Disease

In addition to ACTs, patients with CP are often on inhaled corticosteroids and various bronchodilators at the time of transition, usually with a label of asthma. The presenting patient carried no history of asthma and had been prescribed albuterol and ipratropium as part of her ACT regimen. Medical record review of a clinic-based sample of 452 young adults with CP depicted a higher prevalence of asthma than those without CP.^34^ However, this was based on billing coding, and to our knowledge, there is not robust literature to support that asthma is more common in CP than in the general population.^13^^,^^18^ Patients may be misdiagnosed with asthma because of the presence of wheeze, but there are other causes for wheeze, including bronchiectasis, aspiration, upper airway obstruction, or bronchopulmonary dysplasia if born prematurely, that should not be missed.

If a patient carries a diagnosis of asthma, history, response to inhaler treatments, and workup should be carefully reviewed. Obtaining pulmonary function tests (PFTs) may help confirm the diagnosis but may be limited based on cognitive and/or physical impairments, as was the case in the presenting patient. If a diagnosis of asthma is not supported, consider de-escalating medications, especially because inhaled corticosteroids have been associated with increased risk of pneumonia.^35^

Patients with CP are at risk of recurrent lung infections, suppurative lung disease, and bronchiectasis because of the factors previously discussed. Assessment of oral and dental care and vaccinations should not be forgotten. Microbiological data should be reviewed, and if unavailable repeat sputum cultures may be needed. Chronic *Pseudomonas* *aeruginosa* infections are associated with accelerated decline in pulmonary function, increased hospitalizations, and reduced quality of life.^36^ A small study of three patients with CP and recurrent pneumonias found a reduction in the number of hospitalizations from pneumonias using a regimen of intermittent nebulized tobramycin similar to that used in cystic fibrosis.^37^ The role of additional long-term antibiotics including macrolides in CP has yet to be determined.

## Neuromuscular Scoliosis and Restrictive Chest Wall Disease

Muscle weakness, asymmetrical tone, and postural imbalance in CP can lead to scoliosis.^38^ Like other pulmonary complications in CP, the incidence of scoliosis increases with motor impairment severity.^39^ In CP, progression of scoliosis can occur after skeletal maturity.^40^ The presenting patient had undergone spinal stabilization at 12 years of age for scoliosis. Monitoring for progression of spinal and chest wall deformities is important in the transition of adults with CP, and if not available, we consider a chest radiograph to approximate the degree of severity of scoliosis. Unlike idiopathic scoliosis, the classic curve in CP is a long C-shaped curve often associated with thoracic kyphosis leading to restrictive physiology and impaired ventilatory function.^41^^,^^42^ These changes worsen with age-related decreases in chest wall compliance and respiratory muscle weakness, resulting in restriction on PFT.^43^ Vital capacity is a predictor of the development of respiratory failure, but again, formal PFT can be difficult in these patients.^43^ The degree of impaired mobility and spinal curvature are often indicators of respiratory failure. Respiratory failure is uncommon if spinal curvature is < 100 degrees.^43^

Monitoring for hypoventilation is important because invasive and noninvasive ventilation (NIV) have been shown to improve gas exchange, improve respiratory muscle strength and ventilatory drive, reduce pulmonary hypertension, and improve survival in kyphoscoliosis.^44^ Studies specifically in patients with CP with neuromuscular scoliosis are lacking. Similarly, few studies have explored the impact of therapeutic interventions for neuromuscular scoliosis on pulmonary outcomes in adults with CP. Upright positioning is important in optimizing respiratory function.^45^ Spinal bracing has not been shown to improve lung function in neuromuscular scoliosis and can be associated with further reductions in vital capacity.^45^ Surgery aims to halt curve progression and level the pelvis and is generally considered in the pediatric setting. It can improve deformity and health-related quality of life, but limited studies have shown minimal to no improvement in gross motor and respiratory function with a high pulmonary complication rate.^46^

## SDB and Hypoventilation

Up to one-third of children with CP report sleep disturbances compared with 0% to 5% in the general population.^47^, ^48^, ^49^ Sleep disturbances are likely multifactorial in etiology including but not limited to restricted movements, behavioral problems, cosleeping, and SDB. SDB includes OSA, central sleep apnea, and sleep-related hypoventilation. The few studies exploring SDB in children with CP are mainly based on questionnaires, but they have shown SDB to be more common in CP when compared with typically developing children, ranging between 12% and 44%.^48^, ^49^, ^50^ Changes in upper airway muscle tone including laryngeal dystonia and abnormal neuromuscular control predispose patients to OSA. The prevalence of sleep disturbances and OSA in adults with CP is unknown, but OSA is common in the general population, increasing with age, such that SDB must be considered in the transition of patients with CP.

Clinical history and physical examination alone are inadequate to diagnose SDB. In-laboratory polysomnography remains the criterion standard to evaluate for SDB in patients with neuromuscular and cardiopulmonary disease, especially in suspected hypoventilation. However, access to sleep laboratories with equipment and staff trained to perform sleep studies in patients with CP with limited mobility and often behavioral and cognitive impairment is limited. Although there may be a role for home sleep apnea testing in these patients, studies are needed. Early planning during transition is important, especially because sleep studies may be necessary for ongoing payment of positive airway pressure (PAP) devices and supplies.

CPAP is the mainstay of treatment for adults with OSA, and in a small study of 19 children with CP and OSA, treatment with either adenotonsillectomy or CPAP improved symptoms, daytime functioning, and caregiver concern.^51^ NIV may have additional benefits over CPAP in patients with CP and muscle weakness, restrictive chest wall disease, and reduced ventilatory drive by augmenting ventilation as previously discussed. PAP is not without risk. Adherence to PAP can be challenging. Uncontrolled upper airway secretions and frequent need of suctioning and uncontrolled seizures can hinder success. Of 21 children with CP initiated on NIV for moderate to severe SDB, 55% were intolerant or unable to adhere compared with 8.7% in the total population, most commonly because of intolerance of mask/ventilation pressure.^47^ Among those successfully established on NIV, SDB significantly improved. The presenting patient reported adherence and clinical improvement with NIV, and review of download data from her PAP unit supported control of her OSA on her current settings with minimal leak using a nasal mask. Nasal masks are usually preferred for patients at risk of aspiration and those who cannot remove the mask unassisted.

## Conclusions

The case highlights common pulmonary complications seen in patients with CP and some of the challenges adolescents with CP face when transitioning care from pediatric to adult medicine. It also highlights the challenges adult pulmonologists face caring for these patients with often limited multidisciplinary resources, little quality research on this topic, and minimal to no training on identifying and managing pulmonary complications in this patient population. We hope this review is only a start to tackling some of these challenges.

## Financial/Nonfinancial Disclosures

None declared.
